# Prenatal management and perinatal outcome in giant placental chorioangioma complicated with hydrops fetalis, fetal anemia and maternal mirror syndrome

**DOI:** 10.1186/1471-2393-12-72

**Published:** 2012-07-28

**Authors:** Lutgardo García-Díaz, Práxedes Carreto, Susana Costa-Pereira, Guillermo Antiñolo

**Affiliations:** 1 Unidad de Gestión Clínica de Genética, Reproducción y Medicina Fetal. Instituto de Biomedicina de Sevilla (IBIS), Hospital Universitario Virgen del Rocío/CSIC/Universidad de Sevilla, Sevilla, Spain; 2 Servicio de Obstetricia y Ginecologia, Hospital Juan Ramón Jiménez, Huelva, Spain; 3 Centro de Investigación Biomédica en Red de Enfermedades Raras (CIBERER), Sevilla, Spain; 4 Director de la Unidad de Gestión Clínica de Genética, Reproducción y Medicina Fetal, Hospital de la Mujer, Hospital Universitario Virgen del Rocío, Avda. Manuel Siurot s/n, 41013, Sevilla, Spain

**Keywords:** Fetal chorioangioma, Hydrops fetalis, Fetal anemia, Fetal therapy, Mirror syndrome

## Abstract

**Background:**

Giant placental chorioangiomas have been associated with a number of severe fetal complications and high perinatal mortality.

**Case presentation:**

We report a case of giant chorioangioma with fetal hydrops, additionally complicated by severe anemia, mild cardiomegaly with hyperdinamic heart circulation and maternal mirror syndrome. Intrauterine blood transfusion and amniodrainage was performed at 29 weeks. Worsening of the fetal and maternal condition prompted us to proceed with delivery at 29 + 5 weeks. The newborn died 3 hours later due to pulmonary hypoplasia and hemodynamic failure. Maternal course was favourable, mirror syndrome resolved in the second day and the patient was discharged four days following delivery.

**Conclusions:**

In the case described here, fetal condition got worse despite of the anemia correction and amniodrainage. Our outcome raises the issue whether additional intrauterine clinical intervention, as intersticial laser, should have been performed to stop further deterioration of the fetal condition when progressive severe hydrops develops.

## Background

Chorioangiomas are benign placenta tumors histologically corresponding either to hamartomas derived from primitive chorionic mesenchyma or placental hemangiomas arising from chorionic plate [1]. Large or giant chorioangiomas, defined as measuring more than 4–5 cm in diameter, have an estimated prevalence varying from one in 9000 to one in 50 000 pregnancies [1], and have been associated with a number of fetal complications including anemia, polyhydramnios, hyperdynamic circulation and cardiomegaly, hydrops, and growth restriction [2-6]. In view of these complications and the associated high perinatal death rate (30–40%), a number of therapeutic interventions have been attempted with limited success in most cases [5,7,8].

Here we report a case of giant chorioangioma, fetal hydrops, additionally complicated by severe anemia, mild cardiomegaly with hyperdinamic heart circulation and maternal mirror syndrome.

## Case presentation

**Figure 1:**
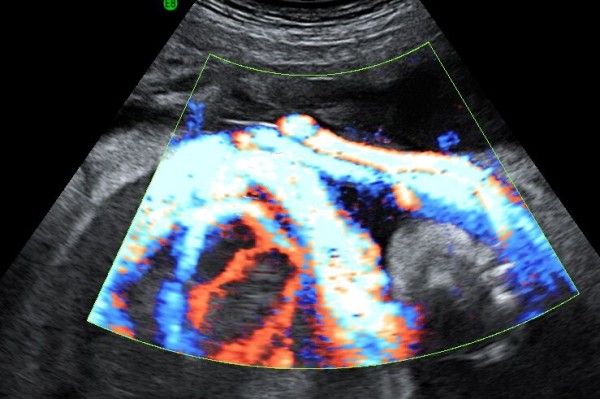
Color Doppler of giant placental choriangioma.

**Figure 2:**
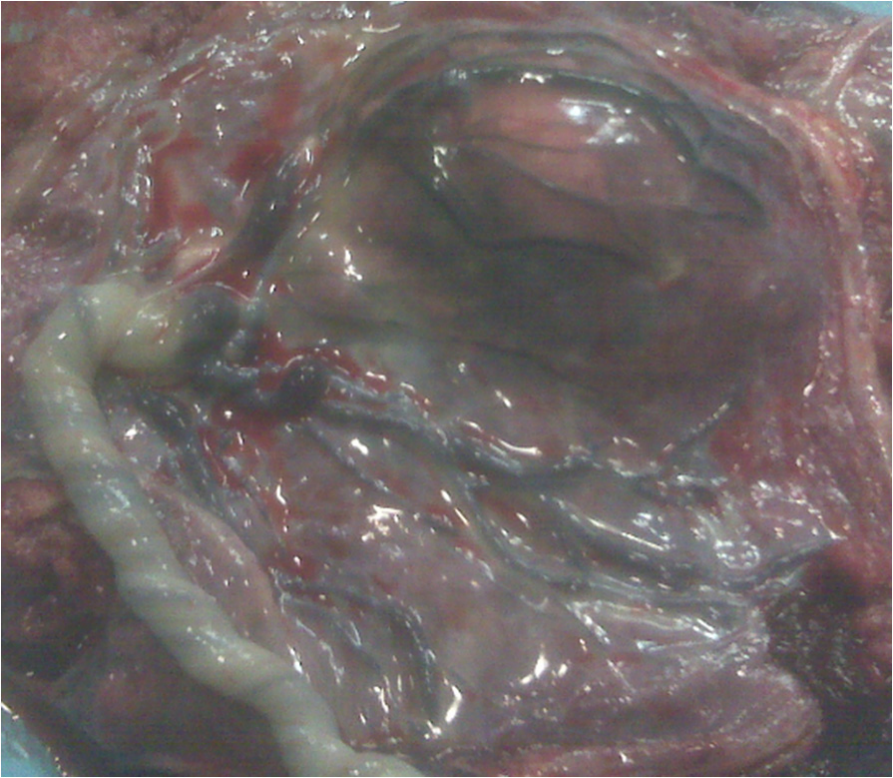
Macroscopic view of giant placental choriangioma.

A 34-year-old woman, gravida 1, was referred to our Department at 29 weeks´ gestation because of placental chorioangioma, severe hydrops fetalis, suspected fetal anemia and maternal mirror syndrome (Ballantine’s syndrome), previously not detected. Ultrasound examination confirmed fetal hydrops with hydrothorax and ascitis, fetal anemia (middle cerebral artery peak systolic velocity: 74.3 cm/sg with an estimated haemoglobin of 7.14 g/dl), polyhydramnios (maximum pocket 14), estimated fetal weight of 2460 g and mild cardiomegaly (cardiac area more than 1/3 of thoracic area) with hyperdinamic heart circulation. In addition, ultrasound examination showed the presence of a heterogenous hypoechoic area of 70x56 mm, with color Doppler showing a specific blood supply, both consistent with diagnosis of chorioangioma (Figure1). Maternal examination was consistent with mirror syndrome (edema, oliguria, anemia, elevated liver enzymes, hypoproteinemia and hypokalemia). Blood pressure was normal. At 29 + 1 weeks, treatment with 12 mg betamethasone injections (two doses separated in 24hours) was given; intrauterine transfusion (IUT) of 80 ml of packed red blood cell suspension with a 70% hematocrit was performed, and followed by amniodrainage. Initial fetal hematocrit was 7 g/dL and final one was 12 g/dL. 1800 ml of amniotic fluid were drained, reducing maximum pocket to 5 cm. Within the next hour preterm labor developed, therapy with atosiban and nifedipine was initiated and the contractions subsided. At 29 + 5 weeks’ gestation, fetal ultrasound showed worsening of the fetal hydrothorax as well as pulsatility index of the umbilical artery over 95^th^ centile. In addition, worsening of the maternal mirror syndrome (pleural effusion and increasing levels of liver enzymes)was noted. Worsening of the fetal and maternal condition prompted us to proceed with delivery at 29 + 5 weeks’. A thoracocentesis to optimise the neonatal resuscitation was performed in the operating room, and was immediately followed by the caesarean section. The newborn weighed 2503 g and apgar score was 1 and 4 at 1 and 5 min. The newborn died 3 hours later due to pulmonary hypoplasia and hemodynamic failure. Maternal course was favourable, mirror syndrome resolved in the second day and the patient was discharged four days following delivery. Histological analysis confirmed the diagnosis of placental chorioangioma (Figure2). Microscopic examination showed that the chorioangioma was composed of predominantly capillary vascular areas in the fibroid matrix.

## Discussion

Around 50% of large chorioangiomas cases develop fetal and maternal complications that required either elective delivery or intervention for tumor-related effects [9].

Chorioangiomas may act as peripheral arteriovenous shunts, leading to increased cardiac output, cardiomegaly and finally heart failure and hydrops, additionally complicated by fetal anemia in some cases [2-6]. When complications appear late in pregnancy, the best option is delivery. However, complications may appear earlier and delivery may be a problem due to fetal prematurity. Thus, different interventions have been proposed to prevent fetal loss related to large fetal chorioangioma complications. Amniodrainage for alleviating polyhydramnios [3,5,7] and intrauterine transfusions in the presence of fetal anemia are two of the most common therapeutic procedures [7,10-12], although results are favorable, the problem that causes increased peripheral flow through the chorioangioma, it is not solved with amniodrainage or fetal transfusion. For that reason, other approaches have been used to stop vascular supply to the tumor and consequent heart failure. In addition to amniodrainage or fetal transfusion, different techniques have been used as injection of absolute alcohol [13-15], endoscopic laser coagulation [16,17], and interstitial laser therapy [4,7], endoscopic suture with bipolar electrosurgery [18], and microcoil and embucrilate embolization, the last two procedures without survivors [9,13,18]. Table 1 summarize a literature review of therapy (excluding amniodrainage alone) and the presence/absence of hydrops as well as other complications in chorioangioma cases. When analysing the overall results of intraturine interventions, it is also remarkable that mortality in cases without fetal hydrops is 10%, while mortality in cases with fetal hydrops raises to 67%. In fact, all successful intersticial laser procedures [4,7] were performed to prevent the development of fetal hydrops.

**Table 1:** Literature review of therapy (excluding amniodrainage alone), presence/absence of hydrops, and other complications in giant chorioangioma cases

**Reference**	**Case #**	**Greatest tumor diameter (mm)**	**Hydrops**	**Other complications**	**Intrauterine therapy**	**Delivery (weeks)**	**Pregnancy outcome**
This report	1	78	Yes	Polyhidramnios, hydrothorax, mild cardiomegaly, fetal anemia, mirror syndrome	Intrauterine transfusion + amniodrainage (29 weeks)	29+6	Neonatal death
Zanardini (2010)	2	42	No	Polyhydramnios and cardiomegaly	Fetoscopic laser (24+3 weeks)	36+3	Live birth
Zanardini (2010)	3	45	No	Moderate cardiomegaly	Interstitial laser (25+4 and 26+4 weeks)	32+3	Live birth
Zanardini (2010)	4	35	No	Mild cardiomegaly and fetal anemia	Interstitial laser (32+3 weeks)	39+1	Live birth
Zanardini (2010)	5	54	No	Mild cardiomegaly and fetal anemia	Amniodrainage (28+6 weeks)	37+3	Live birth
					Intrauterine blood transfusion (29 weeks)		
					Interstitial laser (29 and 30+4 weeks)		
Sisvali (2009)	1	70	Yes	Fetal anemia	Intrauterine blood transfusion at 26	27	Live birth
Mendez-Figueroa (2009)	1	43	Yes	Fetal anemia	Amniodrainage	26	Fetal demise
					Intrauterine blood transfusion		
					Fetoscopy: bipolar and laser coagulation		
Bermúdez (2007)	1	61	Yes	Fetal anemia	Intrauterine blood transfusion.	27	Fetal demise
					Fetoscopic laser coagulation Amniodrainage		
Deren (2007)	1	83	Yes	Fetal anemia	Intrauterine blood transfusion.	28	Live birth
					Alcohol injection into the tumor (25 and 26 weeks)		
Quarello (2005)	1	44	Yes	No	Fetoscopic laser coagulation Amniodrainage	39	Live birth
Escribano D (2005)	1	81	No	Fetal anemia	Intrauterine blood transfusion (25 weeks, 60 ml)	39	Live birth
Lau (2005)	1	90	Yes	Polyhydramnios	Ultrasound guided transcutaneous embolisation with enbucrilate (24+2 weeks)	26	Neonatal death
Lau (2003)	1	74	No	Fetal anemia	Intrauterine blood transfusion (24^th^ weeks, 50 ml)	29 +6	Neonatal death
					Ultrasound-guided transcutaneous embolisation with microcoil (24+2th weeks, 8 pieces)		
					Intrauterine blood transfusion (25^th^ weeks, 60 ml)		
					Ultrasound-guided transcutaneous embolisation with microcoil (25th weeks, 9 pieces)		
					Intrauterine blood transfusion (27^th^ 28^th^ 29th weeks		
Sepúlveda (2003)	1	75	Yes	Severe polyhydramnios and cardiac faillure	Alcohol ablation (26 weeks)	26	Fetal demise (26 weeks)
Nicolini (1999)	1	60	No	Polyhydramnios	Amniodrainage	N/A	Live birth
					Alcohol ablation (27 weeks)		
Nicolini (1999)	2	50	No	Polyhidramnios	Alcohol ablation (24 and 25 weeks)	N/A	Live birth
Haak (1999)	1	68	No	Fetal anemia Polyhidramnios	Intrauterine blood transfusion (30 weeks, 100 ml)	32	Live birth
Quintero (1996)	1	85	Yes	Fetal anemia	Fetoscopy and devascularisation by suture ligation and bipolar cautery (24^th^ weeks)	24+3	Fetal demise

Mirror syndrome (Ballantyne’s syndrome) is usually defined as maternal edema associated to fetal hydrops [19]. Different fetal conditions have been related to mirror syndrome, although pathogenesis and pathofisiology of Ballantyne’s syndrome is currently unknown [19]. Mirror syndrome associated to large placental chorioangiomas has been described only a few times [20-23] and maternal edema has been always present, as was in our case. In addition, other clinical markers also been reported as oliguria, anemia, elevated liver enzymes, hypoproteinemia and hypokalemia were also present in our patient. Additional clinical signs and symptoms described in Mirror syndrome related to large chorioangiomas such as elevated blood pressure, proteinuria, elevated uric acid and creatinine, headache and visual disturbances, and low platelets were absent in our case, which made easier differential diagnosis with preeclampsia. As described elsewhere [19] mirror syndrome disappears shortly after fetal hydrops successful treatment, pregnancy termination or delivery, as in the patient presented here.

## Conclusions

In the case described here, which presented with fetal anemia and severe hydrops, additionally complicated by maternal mirror syndrome, intrauterine transfusion and amniodrainage were performed. However, fetal condition got worse despite of the anemia correction. Finally, an emergency cesarean section, after thoracocentesis to optimise the neonatal resuscitation, was performed due to worsening of maternal and fetal condition and to prevent fetal demise. Current data and experience from clinical practice are still scanty to support the effectiveness of intrauterine therapy procedures in chorioangioma complicated cases, specially in cases with fetal hydrops, which led us to attempt a more conservative approach. However, our outcome raises the issue whether additional intrauterine clinical intervention, as intersticial laser, should have been performed to stop further deterioration of the fetal condition when progressive severe hydrops develops.

## Consent

Written informed consent was obtained from the patient for publication of this report and any accompanying images, as it is our usual publication policy according to our Internal Review Board instructions.

## Competing interests

The authors declare that they have no competing interests.

## Authors’ contributions

GA and LG-D drafted the manuscript, and PC and SC-P collaborated with valuable contributions to the manuscript. All authors have read and approved the final manuscript.

## Pre-publication history

The pre-publication history for this paper can be accessed here:

http://www.biomedcentral.com/1471-2393/12/72/prepub

## References

[B1] Fox H Sebire NJ Fox H, Sebire N Non-trophoblastic tumors of the placenta Pathology of the Placenta 2007 3 Philadelphia: Saunders Elsevier 401 430

[B2] Sepulveda W Aviles G Carstens E Corral E Perz N Prenatal diagnosis of solid placental masses; the value of color flow imaging Ultrasound Obstet Gynecol 2000 16 554 558 10.1046/j.1469-0705.2000.00245.x 11169350

[B3] Sepulveda W Alcalde JL Schnapp C Bravo M Perinatal outcome after prenatal diagnosis of placental chorioangioma Obstet Gynecol 2003 102 1028 1033 10.1016/S0029-7844(03)00859-7 14672481

[B4] Bhide A Prefumo F Sairam S Carvalho JS Thilaganathan BT Ultrasound-guided interstitial laser therapy for the treatment of placental chorioangioma Obstet Gynecol 2003 102 1189 1191 10.1016/S0029-7844(03)00706-3 14607052

[B5] Nicolini U Zuliani G Caravelli E Fogliani R Poblete A Roberts A Alcohol injection: a new method of treating placental chorioangiomas Lancet 1999 353 1674 1675 10335791 10.1016/S0140-6736(99)00781-3

[B6] Jauniaux E Kadri R Donner C Rodesch F Not all chorioangiomas are associated with elevated maternal serum alphafetoprotein Prenat Diagn 1991 11 73 74 10.1002/pd.1970120115 1372974

[B7] Zanardini C Papageorghiou A Bhide A Thilaganathan B Giant placental chorioangioma: natural history and pregnancy outcome Ultrasound Obstet Gynecol 2010 35 332 336 10.1002/uog.7451 19859897

[B8] Quintero RA Reich H Romero R Johnson MP Goncalves L Evans MI In utero endoscopic devascularization of a large chorioangioma Ultrasound Obstet Gynecol 1996 8 48 52 10.1046/j.1469-0705.1996.08010048.x 8843620

[B9] Wehrens X Offermans JPM Snijders M Peeters L Fetal cardiovascular response to large placental Chorioangiomas J Perinat Med 2004 32 107 112 10.1515/JPM.2004.020 15085884

[B10] Sivaslı E Tekşam Ö Haliloğlu M Güçer S Orhan D Gürgey A Tekinalp G Hydrops fetalis associated with chorioangioma and thrombosis of umbilical vein Turk J Pediatr 2009 51 515 518 20112613

[B11] Haak MC Oosterhof H Mouw RJ Oepkes D Vandenbussche FPHA Pathophysiology and treatment of fetal anemia due to placental chorioangioma Ultrasound Obstet Gynecol 1999 14 68 70 10.1046/j.1469-0705.1999.14010068.x 10461342

[B12] Escribano D Galindo A Arbués J Puente JM De la Fuente P Prenatal management of placental chorioangioma: value of the middle cerebral artery peak systolic velocity Fetal Diagn Ther 2006 21 489 493 10.1159/000095659 16969001

[B13] Lau TK Yu SCH Leung TY To KF Fung TY Leung TN Prenatal embolisation of a large chorioangioma using enbucrilate BJOG 2005 112 1002 1004 10.1111/j.1471-0528.2005.00567.x 15958010

[B14] Wanapirak C Tongsong T Sirichotiyakul S Chanprapapah P Alcoholization: the choice of intrauterine treatment for chorioangioma J Obstet Gynaecol Res 2002 28 71 75 10.1046/j.1341-8076.2002.00016.x 12078971

[B15] Deren O Ozyuncu O Onderoglu LS Durukan T Alcohol injection for the intrauterine treatment of chorioangioma in a pregnancy with transfusion resistant fetal anemia: a se report Fetal Diagn Ther 2007 22 203 205 10.1159/000098718 17228160

[B16] Bermudez C Luengas O Perez-Wulff J Genatios U Garcia V Guevara-Zuloaga F Quintero RA Management of a placental chorioangioma with endoscopic devascularization and intrauterine transfusions Ultrasound Obstet Gynecol 2007 29 97 98 10.1002/uog.3903 17201009

[B17] Quarello E Bernard JP Leroy B Ville Y Prenatal laser treatment of a placental chorioangioma Ultrasound Obstet Gynecol 2005 25 299 301 10.1002/uog.1848 15736199

[B18] Mendez-Figueroa H Papanna R Popek EJ Byrd RH Goldaber K Moise KJ Jonson A Endoscopic laser coagulation following amnioreduction for the management of a large placental chorioangioma Prenat Diagn 2009 29 1277 1278 10.1002/pd.2400 19918962

[B19] Braun T Brauer M Fuchs I Czernik C Dudenhausen J Henrich W Sarioglu N Mirror Syndrome: A Systematic Review of Fetal Associated Conditions, Maternal Presentation and Perinatal Outcome Fetal Diagn Ther 2010 27 191 203 10.1159/000305096 20357423

[B20] Dorman SL Cardwell MS Ballantyne syndrome caused by a large placental chorioangioma Am J Obstet Gynecol 1995 173 1632 1633 10.1016/0002-9378(95)90666-5 7503218

[B21] Gherman RB Incerpi MH Wing DA Goodwin TM Ballantyne syndrome: is placental ischemia the etiology? J Matern Fetal Med 1998 7 227 229 9775990 10.1002/(SICI)1520-6661(199809/10)7:5<227::AID-MFM3>3.0.CO;2-I

[B22] Galimberti A Jain S Placental chorioangioma as a cause of maternal hydrops syndrome J Obstet Gynaecol 2000 20 91 10.1080/01443610063633 15512484

[B23] Zoppini C Acaia B Lucci G Pugni L Tassis B Nicolini U Varying Clinical Course of large Placental Chorioangiomas. Report of 3 cases Fetal Diagn Ther 1997 12 61 64 10.1159/000264430 9101227

